# Cultural Adaptation and Psychometric Properties of the Spanish Version of the Occupational Balance Questionnaire: An Instrument for Occupation-Based Research

**DOI:** 10.3390/ijerph18147506

**Published:** 2021-07-14

**Authors:** Paula Peral-Gómez, Sofía López-Roig, María Ángeles Pastor-Mira, Ester Abad-Navarro, Desirée Valera-Gran, Carita Håkansson, Petra Wagman

**Affiliations:** 1 Department of Pathology and Surgery, Miguel Hernández University, 03550 Alicante, Spain; pperal@umh.es (P.P.-G.); eabad@umh.es (E.A.-N.); 2 Grupo de Investigación en Terapia Ocupacional (InTeO), Miguel Hernández University, 03550 Alicante, Spain; 3 Department of Health Psychology, Miguel Hernández University, 03550 Alicante, Spain; slroig@umh.es (S.L.-R.); mapastor@umh.es (M.Á.P.-M.); 4 Department of Occupational and Environmental Medicine, Lund University, 22363 Lund, Sweden; carita.hakansson@med.lu.se; 5 Department of Rehabilitation, School of Health and Welfare, Jönköping University, 55111 Jönköping, Sweden; petra.wagman@ju.se

**Keywords:** occupational balance, questionnaire, adaptation, validation, psychometric testing

## Abstract

Occupational balance (OB) may be a major determinant of health outcomes due to its role in bringing a sense of purpose in the occupations that are personally experienced as a process of getting health and wellness. The Occupational Balance Questionnaire (OBQ) is a reliable instrument for measuring OB, although it has not been adapted and validated in Spain so far; therefore, this study had a double aim: (1) to translate and cross-culturally adapt the original OBQ version into Spanish (OBQ-E); (2) to analyze the psychometric properties for its use in the Spanish population. Standard procedures were used for the cross-adaptation process and pilot testing was carried out using three different samples to extend the applicability of the OBQ-E. Validation measures of the final version of the OBQ-E were conducted in a sample of 219 participants. The OBQ-E showed that items and instructions were culturally appropriate and written clearly. Psychometric testing showed excellent internal consistency (Cronbach’s alpha = 0.87; Guttman split-half coefficient = 0.85), good test–retest reliability (r_s_ (Spearman rho) = 0.73), and acceptable convergent validity (overall status, r_s_ = 0.37; Related Quality of Life, r_s_ = 0.42; Satisfaction with Life Scale, r_s_ = 0.54). The findings suggest that the OBQ-E may be a suitable instrument for assessing OB in the Spanish population; hence, it is a promising tool for epidemiological research that will significantly contribute to the understanding of OB as a health-related factor. Nevertheless, further investigation is also warranted to explore the potentiality of this instrument for clinical purposes.

## 1. Introduction

Occupational therapy practice is based on the fundamental assumption that meaningful occupations play a key role in achieving and maintaining health and wellbeing by facilitating personal engagement and accomplishment [1]. This practice framework rests largely on the fact that occupational performance should involve an occupational balance (OB), i.e., a sense of harmony between occupations, becoming a necessary condition to ensure that occupations properly perform a process for obtaining health and wellness [2]. From an occupational perspective of health, it should be noted that there is a variety of risk factors that can affect the right use, choice, opportunity, or balance in an occupation, thereby resulting in occupational dysfunction, which may also involve other negative consequences such as occupational deprivation, occupational alienation, or occupational imbalance [3]. In this respect, it is important to have accurate instruments for identifying potential occupational determinants in order to examine their effects on health outcomes; however, the number of available instruments with which to perform occupation-based research remains low.

As a core concept for occupational therapy practice, OB should be considered an important area of research, although its development is still at an incipient stage and it is a relatively new study area. To date, several studies have provided some evidence that OB may be a major occupational determinant of health outcomes. On a positive note, the main research findings have suggested that OB is perceived as a good proxy of self-rated health [2,4,5,6,7,8] and related to life satisfaction [2,4,5,6] and health-related quality of life [2,9]. On the other hand, some studies have reported that occupational imbalance is linked to perceived stress [2,5,10,11].

Although it is recognized that OB is a very complex term to assess, the literature suggests three different forms of OB as main indicators to measure this complicated subjective experience. Along with maintaining a “harmonic mix” within occupations, OB has been conceptualized as the capacity to manage the amount and variation of duties within an occupation while preserving personal preferences, as well as the ability to maintain a strong sense of self-identity through participation in meaningful occupations based on personal values [12]. To our knowledge, there are 23 different instruments for assessing OB [13,14,15,16]. Most of these instruments were initially designed for clinical purposes and not as assessment tools for research. In fact, the available research on OB is based largely on the use of several instruments that were recently developed: the Occupational Balance Questionnaire (OBQ) [14], the Occupational Value with Predefined Items (OVal-pd) [17], the Satisfaction with Daily Occupations and Balance (SDO-OB) [15], and the Occupational Balance-Questionnaire (OB-Quest) [16]. The OBQ [14] particularly focuses on assessing the person’s satisfaction with the amount and variation of occupations, resulting in a global picture of one’s own occupational balance. Unlike the other OB instruments, this questionnaire examines people’s own perceptions of their OB, with the advantage of representing a wide variety of everyday occupations rather than focusing on a single category (e.g., housework, rest, work, etc.) [14]. Interestingly, this questionnaire was developed using a holistic approach based on the experiences of occupations as a way of integrating the three dimensions of OB within a broader scope, thereby providing a full assessment of a person’s occupational performance. It consists of 13 items that measure the current experience of OB according to the quantity of and variability between the occupations and their significance within one’s own personal occupational pattern. Each item can be rated on a scale of 0 (i.e., completely disagree) to 5 (i.e., completely agree). The total score can be obtained by calculating the sum of the respective values of each item, ranging from 0 to 65 points. The OBQ has shown a good internal consistency (Cronbach alpha, 0.94) and test–retest reliability (r_s_ (Spearman rho) = 0.93) [18], which makes it a reliable instrument to measure OB.

The development of the Spanish version of the OBQ constituted a central part of the doctoral thesis done by P.P.-G. To our knowledge, this version has been used in several previous studies aimed at examining OB in Spanish adults [19,20,21]; therefore, publishing the results of the adaptation and validation of the instrument is crucial to enhancing the accuracy of the research focused on OB in the Spanish population. The present study had a double aim: (1) to translate and cross-culturally adapt the original OBQ version into Spanish (OBQ-E); (2) to analyze the psychometric properties of the OBQ-E for use in the Spanish population.

## 2. Materials and Methods

### 2.1. Study Design

**Figure 1:**
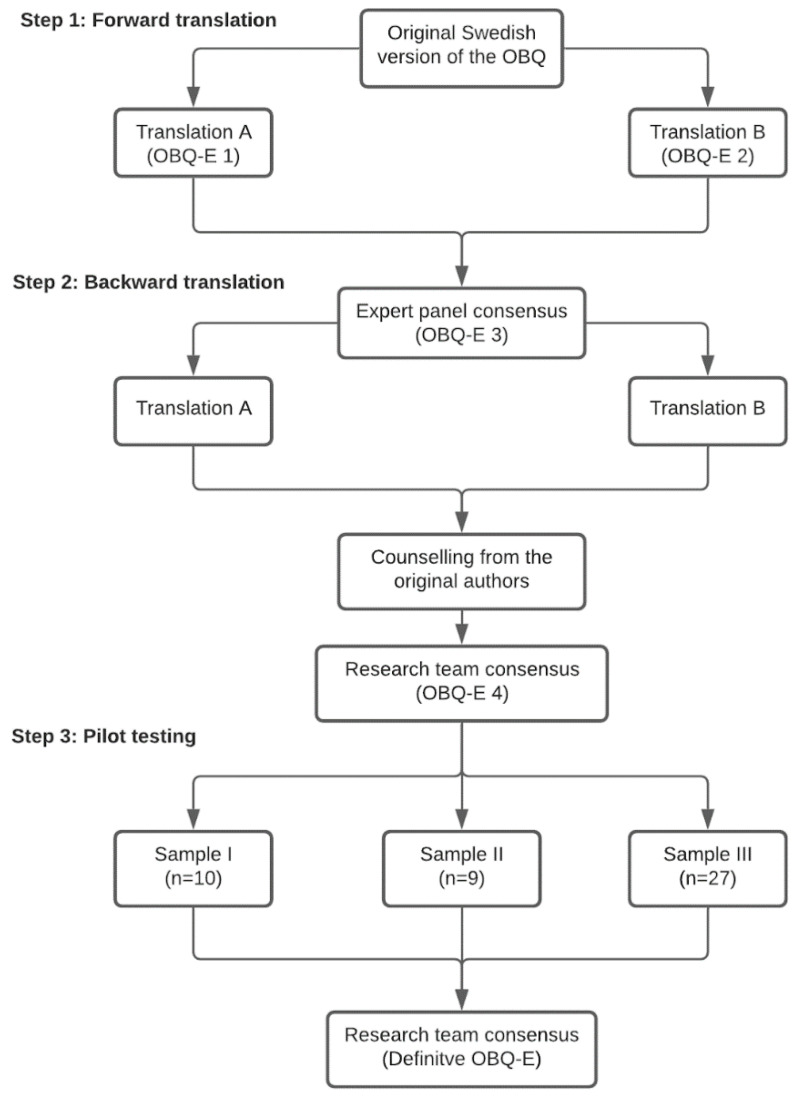
Flow chart for the cross-cultural adaptation and translation process of the OBQ-E.

The present study was carried out in two phases. In the first phase, a linguistic validation of the Spanish version of the OBQ (i.e., OBQ-E) was conducted through a cross-cultural adaptation and dual forward–back translation of the original text. In the second phase, a psychometric testing of the OBQ-E was performed to verify that the items of the Spanish version measured what they were intended to measure (Figure 1). The study was approved by the ethics committee of the Miguel Hernández University (DCP.PPG.01.16) and the research was performed in accordance with the Declaration of Helsinki. All subjects participated voluntarily and signed an informed consent form. The processing, communication, and transfer of personal data of the participants was in accordance with the provisions of Law 15/99 of 13 December on the Protection of Personal Data.

### 2.2. Participants and Recruitment

To perform the linguistic validation of the questionnaire, we recruited three different samples of participants (*n* = 46) to widen the scope of the applicability of the OBQ-E (Table 1). Sample I (*n* = 10) consisted of patients with fibromyalgia, without cognitive impairment or literacy difficulties, who had different cultural backgrounds. They were recruited as representatives of people with chronic health problems and as potential users of occupational therapy services in order to extend the conceptual and linguistic equivalence to people with medical conditions. Sample II (*n* = 9) included students in the final year of their bachelor’s degree in occupational therapy in the Miguel Hernández University (Alicante, Spain). Sample III (*n* = 27) consisted of occupational therapy graduates.

To evaluate the psychometric properties of the Spanish final version of the instrument obtained from the earlier cross-cultural adaptation and translation process, a total of 219 undergraduate occupational therapy students (sample IV) from Miguel Hernández University were also enlisted (Table 1).

### 2.3. Instruments

The Occupational Balance Questionnaire (OBQ) [14] is a short 13-item self-report questionnaire for measuring OB that takes approximately 5 min. Respondents are requested to use a 6-point ordinal scale ranging from 0 (completely disagree) to 5 (completely agree) to give their appraisal of each statement. Higher scores indicate better OB, while the total maximum score that can be obtained in the OBQ-E is 65 by adding up the points for each item.

The Global Health Status/Quality of Life (GHS/QoL) is a subscale included in the Quality of Life Questionnaire (EORTC QLQ-C30, version 3) and is a general quality of life instrument for cancer patients consisting of 30 questions [22]. This questionnaire has also been used in the general population to generate normative data across 15 countries, including the Spanish population [23]. The GHS/QoL subscale comprises two items—one for overall health status and one for health-related quality of life, which can be scored on a Likert-type response scale of 7 points, ranging from 1 (“poor”) to 7 (“excellent”). Higher scores on this subscale indicate a better global health status. The validated Spanish version of this instrument has shown good psychometric properties [24]. Internal consistency showed a Cronbach alpha > 0.70 and validity measures displayed correlation coefficients of 0.4 or higher.

The Satisfaction With Life Scale (SWLS) is a self-reported questionnaire originally created by Deiner, Emmom, Larsen, and Griffin (1985) [25]. It assesses life satisfaction based on five items, rated on a scale of 1 (totally disagree) to 7 (totally agree) points. In this study, we used the validated Spanish version of SWLS, in which the response option was reduced to a scoring range from 1 (totally disagree) to 5 (totally agree) points. As such, the total scores can be between 5 and 25 points, and higher scores indicate greater life satisfaction. The Spanish version of the questionnaire had good internal consistency (Cronbach alpha 0.84) and adequate construct validity (correlation coefficients between 0.30 and 0.50 [26].

### 2.4. Procedure

#### 2.4.1. First Phase: Cross-Cultural Adaptation and Translation of the OBQ-E

The authors of the original version of the OBQ were asked to collaborate in the adaptation and translation of the scale for counselling on all the aspects related to conceptual adaptations of the test. The forward- and back-translation process was carried out through the following steps (Figure 1):

Step 1: Forward translation. Two independent bilingual Spanish professional translators translated the original questionnaire from Swedish into Spanish, generating two different versions. An expert panel (one medical doctor, two psychologists, two occupational therapists, and one professional translator) compared the two translations to check their semantic and cultural appropriateness and whether the original meaning was maintained, resulting in a first Spanish version of the questionnaire.

Step 2: Backward translation. Two bilingual Swedish translators made two independent back translations of this first Spanish version of the OBQ. The authors of the original questionnaire compared these two back translations with the original version to assess potential differences. After the research team discussed and resolved the discrepancies by introducing semantic and idiomatic changes and conceptual adaptation when necessary, the pre-final Spanish version of the OBQ was accepted.

Step 3: Pilot study. The pre-final version of the instrument was tested in a pilot study with 46 subjects selected from samples I, II, and III. In addition to the administration of the questionnaire, the instructions, response scale, comprehension, and clarity of the items were also evaluated to detect possible difficulties in applying different interview techniques to collect participant comments as follows. After completing the questionnaire, participants from sample I participated in a semi-structured group interview. Those enlisted in sample II filled out the questionnaire individually using a “think aloud” technique [27]. Participants from the sample III responded in writing to different open-ended questions included at the end of the questionnaire. All of the participants’ comments were reviewed and discussed by the expert panel to verify the definite Spanish version of the Occupational Balance Questionnaire, OBQ-E.

#### 2.4.2. Second Phase: Psychometric Testing of the OBQ-E

The internal consistency, convergent validity, and test–retest reliability were the psychometric measures used for the validation of the OBQ-E. On this occasion, the OBQ-E was completed using a convenience sample of 219 occupational therapy students (sample IV). To evaluate test–retest reliability, a subsample of 49 students completed the OBQ-E twice within a time interval of no more than 4 months to ensure that the circumstances and contexts of both measurements were similar. As such, holidays, internships, and examination periods were avoided when setting the date for completing the second test.

### 2.5. Data Analysis

The content validity of the OBQ-E was analyzed using the reports based on the reviews of the different translations of the questionnaire done by the expert panel, as well as by examining the comments about the interpretability of the OBQ-E items made by the participants in the first phase of the study. Using a group consensus method, each item was examined for content, meaning, wording, format, ease of administration, and scoring by the expert panel. Each item was either accepted, rejected, or accepted with modification by consensus. Data from the participants’ comments were analyzed by using the transcribed recordings (sample I and II) and the responses from the open-ended questions (sample III). All data were coded using NVivo software, a qualitative data analysis program. The emerging themes were discussed within the research team and conflicting results were resolved by seeking agreement with the original authors. Finally, the expert panel reviewed all items and modified, added, or deleted any content still considered to be irrelevant or unclear. In addition, a descriptive analysis of each OBQ-E item was carried out to verify that the items measured what they were originally intended to measure and to detect ceiling or floor effects. The presence of ceiling or floor effects was assumed if at least 15% of respondents received the highest or lowest possible score, respectively [28]. We also explored those items that presented responses with values less than 5%.

The internal consistency of the OBQ-E was assessed using the Cronbach alpha coefficient and Guttman split-half coefficient. A Cronbach alpha coefficient of 0.70 or higher is generally considered as an acceptable value for good internal consistency [28]. Similarly, a Guttman split-half coefficient between 0.80 and 0.90 is normally seen as a highly reliable value for research instruments [29]. Convergent validity and test–retest reliability were estimated using Spearman correlation coefficients. A coefficient of 0.50 or higher was considered as a strong Spearman correlation [30].

All statistical analyses were conducted with R statistical software version 4.0.0. (R Foundation for Statistical Computing, Vienna, Austria; http://www.r-project.org. Statistical significance was established at a *p* value ≤ 0.05 and a 95% confidence level.

## 3. Results

### 3.1. Cross-Cultural Adaptation, Translation, and Content Validity of the OBQ-E

#### 3.1.1. Report on the Forward Translation

The main results of the translation of the OBQ into Spanish were summarized in a report. Briefly, there were discrepancies in most of the items between the two Swedish-to-Spanish translations of the OBQ, although they were mainly due to the use of different words to express the content of the item (e.g., the expressions “I have balance” or “I maintain balance” in items 1, 4, 8, and 12). Regarding idiomatic issues, the equivalent expressions “I make enough variety”, “I have enough variety”, “I vary sufficiently”, and “I have enough variation” used for the translation of the items 5 and 11 were considered as confusing and unusual in Spanish, opting instead for “I vary enough” as a more suitable expression. Moreover, regarding semantic issues, one of the major discrepancies arose from the translation of the terms “energy-giving activities” and “energy-taking activities” included in item 12 into the expressions “passive and active activities” and “sedentary and physical activities”. The authors of the original questionnaire clarified the meaning of these terms and provided examples to improve their understanding and conceptual equivalence. As a result, considering that energy-giving activities do not necessarily have to be passive and that energy-taking activities are not always active, the original terms were maintained as being the most appropriate.

#### 3.1.2. Report on the Backward Translation

A report compiling the main results of the translation of the first Spanish version of the OBQ into Swedish was also made. Overall, problems of semantic congruence and conceptual equivalence were the main reasons for discrepancy when comparing the two back translations of the OBQ. For example, the items that contained statements beginning with “I have” (i.e., items 1, 4, 8, and 12) were changed to “I maintain”, since the term “maintain” is considered more appropriate than “have” to express balance between different things or aspects in Spanish; however, both terms were included in the pre-final version of the OBQ-E used for the pilot testing to check potential misunderstandings. Moreover, following the technical advice of the authors of the original version, minor changes were made to improve the pre-final version of the questionnaire. The expression “I have enough variety” used in items 5 and 11 of the original version was translated from Spanish as “I vary enough”; however, the authors of the original OBQ explained that these items had to indicate satisfaction and not only variation, so the initial wording (“I have enough variety”) was retrieved in both items (i.e., items 5 and 11).

#### 3.1.3. Pilot Testing of the OBQ-E

For the pilot study, two preliminary Spanish versions of the OBQ were randomly distributed among participants (*n* = 46)—one questionnaire including the expression “I have balance” for items 1, 4, 8, and 12, and one including the expression “I maintain balance” for the same items.

Table 2 summarizes the analysis of comments and proposals provided by participants that helped in elaborating the definitive Spanish version of the OBQ, i.e., OBQ-E. In general, the contents of the items and instructions were well understood and positively valued by the participants. Regarding the items containing the expressions “I have balance” or “I maintain balance” for items 1, 4, 8, and 12, no differences were found in the way these were understood. At the suggestion made by the participants who were occupational therapy graduates, the term “study” was included as an everyday activity in item 4 to differentiate it from “work”. This everyday occupation (i.e., study) was not included in the original version. In summary, after discussing all participant comments and proposals, the research team opted to make a few slight modifications to the final version of the OBQ-E. In this version, the instructions at the beginning of the questionnaire provide a more precise definition of the term “occupation” and a brief explanation of why all the items should be completed, even if some items seem to be somewhat similar to each other. In addition, the wording of the OBQ-E items was improved in order to clarify that everyday activities should be understood as generic terms. The final version of the OBQ-E was carefully reviewed by the research team to check that statements were idiomatically and semantically well-constructed and to ensure the items reflected an adequate level of cultural understanding. This version preserves the structure (i.e., 13 items) and scoring system (i.e., a six-point response scale) from the original version.

### 3.2. Psychometric Testing of the OBQ-E

#### Internal Consistency, Test–Retest Reliability, and Convergent Validity

The results of psychometric measures of OBQ-E were computed in a sample of 219 undergraduate occupational therapy students (sample IV). The Cronbach’s alpha coefficient of the OBQ-E items was 0.87, ranging from 0.85 to 0.87 when one of the items was removed from the test, indicating good internal consistency. Similarly, the Guttman split-half coefficient was 0.85 (part 1, Cronbach’s alpha = 0.72; part 2, Cronbach’s alpha = 0.82). The results of the test–retest reliability of the total OBQ-E showed a strong Spearman correlation coefficient of 0.73 (*p* < 0.001).

Table 3 displays the score distribution for the total OBQ-E, GHS/QoL (overall status and related QoL), and SWLS, while Spearman correlation coefficients were estimated for convergent validity. As displayed, the total OBQ-E was positively correlated with GHS/QoL (both overall status and related QoL), showing acceptable correlation values of 0.37 (*p* < 0.001) and 0.42 (*p* < 0.001), respectively, and with SWLS r_s_ = 0.54, *p* < 0.001, which indicated a strong correlation. Regarding the response distribution for the total OBQ-E score, no ceiling or floor effect was detected. With respect to each item, the percentage of cumulative responses in the maximum score value (i.e., ceiling effect) was less than 12.7% for most items, except items 2 (28.8%), 3 (20.6%), and 6 (25.1%). The percentage of cumulative responses in the minimum score value (i.e., floor effect) was less than 1.4% overall. In six OBQ-E items, no answers with a value of 0 were obtained (items 2, 3, 4, 10, 12, and 13). Item 6 had no responses with values of 0 and 1 points.

## 4. Discussion

This study had a double purpose: first, to translate and cross-culturally adapt the OBQ for use in the Spanish population (first phase); second, to analyze the psychometric properties of the Spanish version of the OBQ (i.e., OBQ-E) (second phase). The findings of the first phase showed that there is a linguistic and conceptual equivalence between the OBQ-E and the original instrument, thereby ensuring good content validity. To improve the clarity and comprehensibility of the final version of the OBQ-E obtained from this first part of the study, a few slight changes were introduced by extending the instructions at the beginning of the questionnaire to facilitate its completion and by rewriting some items using idiomatic expressions or terminology more appropriate to the Spanish context. It should be noted that the cross-cultural adaptation of the questionnaire was conducted in three different samples, including people suffering from fibromyalgia, in order to widen the applicability of the OBQ-E to several segments of the Spanish population. Moreover, the results of the psychometric testing showed good internal consistency, test–retest reliability, and convergent validity, which indicates that the OBQ-E is a valid and reliable instrument for evaluating OB in the Spanish population. To our knowledge, this is the first study ever done to validate a self-reported questionnaire on OB among people from the general population in Spain with a sufficiently large sample size, which reinforces the validity and reliability previously reported for the same questionnaire [14,31].

Although the content validity of the OBQ-E was not assessed by using quantitative measures, e.g., a content validity index (CVI), the panel of experts had the counselling and expertise of the original authors of the questionnaire to help in performing this first evaluation of the instrument during the translation and adaptation process. Moreover, it should be noted that this process was performed according to standardized procedures [32,33] commonly used for the translation and cross-cultural adaptation of self-reported measures such as ours. This process involved a series of steps including forward and backward translation and their respective expert panel’s reviews, as well as the pilot testing of the pre-final version of the questionnaire. This last step entailed the analysis of the content of all the items and instructions (i.e., wording, terminology, scale of answers, etc.) and the participants’ feedback, which were used as performance indicators of the OBQ-E. Importantly, the pilot testing constituted a necessary step to verify that the pre-final version of the OBQ-E was suitable to the target population. All items of the final version were satisfactorily comprehensible and relevant to assess OB and the 6-point scale of answers was deemed appropriate. This suggests that the OBQ-E may adequately represent the concept of OB and seems to show good construct validity. After careful review by the expert panel and due consideration by the authors of the original version, the definitive version of the OBQ-E was approved for further psychometric assessment.

The estimates from the psychometric testing of the OBQ-E were similar to the results obtained from previous validation studies of the only two currently available versions of the OBQ, the original Swedish version [14] and the English adapted version [31]. Tested on a sample of 67 healthy people, the original OBQ presented excellent results for internal consistency (Cronbach’s alpha = 0.94), such as for test–retest reliability (r_s_ for total OBQ score = 0.93), without evidence of ceiling or floor effects [14]. The validation of the English version was estimated in a sample of 86 adults and only provided results of the test–retest reliability (r_s_ = 0.74; *p* = 0.003) [31]. Given that the time interval between the first and second administration of the questionnaire was very different between our study (i.e., 4 months) and the other studies (i.e., a one-week period in the Swedish study and a two-week period in the English study), it could have been reasonably expected that the test–retest coefficient calculated from our data would be lower; however, the estimates remained very close to those found in these studies. Although it may be argued that we did not follow the recommended time interval of 1–4 weeks between the measurements [28,34], it should be noted that the timing of administration can considerably vary because of the type of study variables [34], especially when validating instruments that measure changing variables such as OB. In such cases, the time interval must be a period of time in which it is expected that there will be no changes in the study subjects by ensuring the assessment will be conducted under the same conditions on both occasions [34]; thus, in terms of reproducibility, our test–retest reliability findings verified that the OBQ-E seems to be a stable instrument capable of producing the same results in the same circumstances [28,35]. Regarding the results for convergent validity, our study showed that the total score of the OBQ-E was related to several global measures of health (i.e., overall health status and related quality of life as measured by GHS/QoL) and wellbeing (i.e., satisfaction with life as measured by SWLS). These findings, although not directly comparable, are in line with those observed for the total score for the English version of the OBQ, which was positively related to health status measured by Short Form Health Survey-36 Version 2.0 (SF-36v2) and negatively related to stress as assessed by the Perceived Stress Scale-10 (PSS-10) [31].

The results of this study also showed a ceiling effect for items 2, 3, and 6 of the OBQ-E. In terms of sensitivity, this may suggest that the instrument might not measure or detect small changes in the higher score response options in relation to meaningful activities in daily life, things that someone really wants to do, and enough things to do in a typical week; however, this should not be attributed to a measurement error of the instrument itself, but rather to the fact that the study sample was very homogeneous with very similar sociodemographic conditions. In fact, no ceiling or floor effects were found for the rest of the items or for the total score, suggesting that the questionnaire has adequate sensitivity to assess OB. Nevertheless, we are aware that the OBQ-E should be applied in more heterogeneous populations with diverse sociodemographic situations in order to improve its accuracy as a screening instrument of OB.

This study presents several limitations and strengths that should be acknowledged. While the content validity of the OBQ-E was examined using three different samples to extend its applicability to the Spanish general population, it must be recognized that its external validity was conditioned to a convenience sample, which although was sufficiently large, consisted of university students that were mainly women; however, our analyses were performed using adequate measures and the results coincide with the results obtained from the prior versions of the OBQ, somewhat reinforcing the validity and reliability previously reported for this questionnaire. To overcome the lack of representativeness, this study was enlarged to generate normative values using a wider Spanish population sample with different sociodemographic characteristics (age, gender, and educational level) recruited according to the reference population’s rates published by the National Statistics Institute (INE) of Spain [36]. Moreover, these data will be used to replicate the psychometric evaluation of the OBQ-E by conducting a more extensive analysis, thereby ensuring the generalization of the findings and enhancing the accuracy and capacity of this scale as a screening instrument of OB for the general population. In the meantime, we believe that the estimates of the present study provide convincing evidence to support the accuracy of OBQ-E as a suitable instrument for research, as well as serve as a methodological basis for the incipient epidemiological research focused on OB [19,20,21].

## 5. Conclusions

The OBQ-E may be used as a suitable instrument for assessing OB in the Spanish population. The results of this study show that the Spanish version of the original OBQ has good content validity, moderate convergent validity, strong test–retest reliability, and excellent internal consistency. Judging by the findings from recent studies, the use of this instrument may be of help in monitoring and preventing OB-related problems in the community. For epidemiological research purposes, this is a promising tool that can yield accurate and useful information that will significantly improve the knowledge of OB as a health-related factor. Nevertheless, the potentiality of this instrument for clinical purposes also warrants further investigation.

## Figures and Tables

**Table 1:** Sociodemographic features of the study participants.

Variables	Sample I ^1^ (*n* = 10)	Sample II ^1^ (*n* = 9)	Sample III ^1^ (*n* = 27)	Sample IV ^1^ (*n* = 219)
Age, mean (SD)	47.2 (9.2)	31.6 (7.5)	26.2 (4.0)	22.2 (3.9)
Age range, min.-max.	35–64	23–46	23–37	18–48
Sex, *n* (%)				
Male	1 (10.0)	-	2 (7.4)	23 (10.5)
Female	9 (90.0)	9 (100.0)	25 (99.6)	196 (89.5)
Marital status, *n* (%)				
Single/divorced	2 (20.0)	6 (66.6)	22 (81.5)	207 (94.5)
Spouse/domestic partner	8 (80.0)	3 (33.3)	5 (18.5)	9 (4.1)
Children aged less than 18, *n* (%)				
yes	4 (40.0)	3 (33.3)	1 (3.7)	7 (3.2)
no	6 (60.0)	6 (66.6)	26 (96.3)	206 (94.1)
Professional status, *n* (%)				
Student	-	7 (77.7)	-	174 (79.5)
Student and worker	-	2 (22.2)	-	45 (20.5)
Worker	9 (90.0)	-	17 (63.0)	-
Unemployed	1 (10.0)	-	10 (37.0)	-
Educational level, *n* (%)				
Primary studies	1 (10.0)	-	-	-
Secondary studies	5 (50.0)	-	-	-
University studies	4 (40.0)	9 (100.0)	27 (100.0)	219 (100.0)
Degree in occupational therapy, *n* (%)				
1st year	-	-	-	38 (17.4)
2nd year	-	-	-	59 (26.9)
3rd year	-	-	-	51 (23.3)
4th year	-	9 (100.0)	-	71 (32.4)

Abbreviations: SD, standard deviation. ^1^ Sample I, fibromyalgia patients; sample II, occupational therapy students in their final year; sample III, occupational therapy graduates; sample IV, undergraduate occupational therapy students.

**Table 2:** Analysis of the participant comments and proposals on the final Spanish version of OBQ tested in the pilot study (*n* = 46).

Item Description	Sample I ^1^ (*n* = 10)	Sample II ^1^ (*n* = 9)	Sample III ^1^ (*n* = 27)
1. Balance between doing things for others/for oneself.	Understandable. Proposals: to specify activities (work, leisure, etc.), replace balance with another word.	Understandable. No proposals.	Understandable. No proposals.
2. Perceiving one’s occupations as meaningful.	Understandable. Proposals: to specify activities (work, leisure, etc.).	Understandable. Proposals: to explain what the activities of daily life are.	Understandable. Proposals: to specify more or ask as a question.
3. Time for doing things wanted.	Understandable. Proposals: to specify activity by activity, e.g., “I do the work I really want to do”.	Understandable. Proposals: to specify more.	Understandable. No proposals.
4. Balance between work, home, family, leisure, rest, and sleep.	Understandable. No proposals.	Understandable. No proposals.	Understandable. Proposals: to add the “study”.
5. Balance between doing things alone/with others.	It was understood as either variety between groups of activities or difference between groups. Proposals: to divide the item by types of activity.	It was understood as either variety between groups of activities or difference between groups. No proposals	It was understood as either variety between groups of activities or difference between groups. Proposals: to clarify the comparison by indicating if they are imposed activities or not.
6. Having sufficient to do during a typical week.	Understandable. Proposals: to indicate if it is about leisure activities or obligations.	Understandable. Proposals: to indicate if it is about leisure activities or obligations.	Understandable. Proposals: to clarify the term “sufficient” and specify activities (work, leisure, etc.).
7. Have sufficient time for doing obligatory occupations.	Understandable. Proposals: to specify if activities are general or specific.	Understandable. No proposals.	Understandable. Proposals: to indicate if it refers to obligations or activities that you want to do.
8. Balance between physical, social, mental, and restful occupations.	Understandable. Proposals: to ask as a question considering the time factor.	Understandable. No proposals. The question arises whether all the items must be answered even if they seem similar.	Understandable. Proposals: to include “emotional” activities.
9. Satisfaction with how time is spent in daily life.	Understandable. No proposals.	Understandable. No proposals.	Understandable. No proposals.
10. Satisfaction with the number of activities during a typical week.	Understandable. Proposals: to specify if activities are general or leisure activities.	Understandable. Proposals: to specify if activities are general or leisure activities.	Understandable. No proposals.
11. Balance between obligatory/voluntary occupations.	Understandable. No proposals.	Understandable. Proposal: to include the temporal factor.	Understandable. Proposal: to clarify the term “variety”, make the item more concrete.
12. Balance between energy-giving/energy-taking activities.	Understandable. Proposals: to give concrete examples.	Understandable. Proposals: to give concrete examples.	Understandable. Proposals: to give concrete examples.
13. Satisfaction with time spent in rest, recovery, and sleep.	Understandable. No proposals.	Understandable. No proposals.	Understandable. No proposals.

Abbreviations: OBQ, Occupational Balance Questionnaire. ^1^ Sample I, fibromyalgia patients; sample II, occupation therapy students in their final year; sample III, occupational therapy graduates.

**Table 3:** Score distribution for the total OBQ-E, GHS/QoL (overall status and related QoL), and SWLS, and convergent validity.

Measures	Min.	Max.	Median	P_25_	P_75_	M	SD	r_s_	*p*
OBQ-E	22	65	44	39	49	43.6	7.8	-	-
GHS/HoL									
Overall status	1	7	6	5	6	5.5	1	0.37	<0.001
Related QoL	1	7	6	5	6	5.7	1.1	0.42	<0.001
SWLS	6	25	20	18	22	19.4	3.7	0.54	<0.001

Abbreviations: OBQ-E, Occupational Balance Questionnaire, Spanish version; GHS/QoL, Global Health Status/Quality of Life; Related QoL, Related quality of life; SWLS, Satisfaction With Life Scale; M, mean; SD, standard deviation; r_s_, Spearman correlation coefficient.

## Data Availability

The data presented in this study are available on request from the first author P.P.-G.
